# Critical shoulder angle: a “combined shoulder angle”—balanced contributions of glenoid inclination and acromial angle in shoulder arthritis progression

**DOI:** 10.1016/j.xrrt.2026.100812

**Published:** 2026-06-29

**Authors:** Marc-Olivier Gauci, Hoel Letissier, Christophe Andro, Gilles Walch, Antoine de Chanterac

**Affiliations:** a Universitary Institute for Locomotion & Sport, Orthopaedic unit, Hôpital Pasteur II, Nice, France; b ICARE unit, InsermU1091, IBV, Côte d’Azur University, Nice, France; c Department of Orthopaedic and Trauma Surgery, Hôpital de la Cavale Blanche, Brest, France; d LaTIM, INSERM, UMR 1101, SFR IBSAM, Brest, France; e Service de Chirurgie Orthopédique et Traumatologique, Hôpital d’Instruction des Armées Clermont-Tonnerre, Brest, France; f Clinique Santy, Lyon, France

**Keywords:** Critical shoulder angle, Cuff tear, Glenoid inclination, Shoulder morphometry, Acromial angle, Glenohumeral arthritis, Scapular morphology, Shoulder pathology prediction

## Abstract

**Background:**

The critical shoulder angle (CSA) has been described as a morphometric value that could be predisposing to the evolution of a shoulder toward a primary glenohumeral osteoarthritis (PGHOA) or massive rotator cuff tear (MRCT). CSA is described as the sum of the acromial angle (AA) and the glenoid inclination (GI). The aim of this study is to evaluate the impact of GI and AA measurements on CSA value in PGHOA and MRCT.

**Methods:**

Computed tomography scans of 186 patients were extracted from our database and assigned into 3 groups: control group (48 healthy shoulders) and 2 pathological shoulder groups with no glenoid erosion: 66 MRCT (E0 glenoids) and 72 PGHOA (A1 or B1 glenoids). Computed tomography scans were analyzed using automatic software. The GI, AA, and CSA variables were compared knowing that AA + GI=CSA.

**Results:**

There was a significant difference (*P* < .001) in CSA between the MRCT, PGHOA, and control groups, respectively (33 ± 4°, 29 ± 4°, and 32 ± 4°). There was a significant difference between the MRCT and PGHOA for GI (8 ± 5° and 6 ± 6°, respectively, *P* = .026) and no difference with the control group (7 ± 5°, *P* > .05). The AA was not statistically different among the 3 groups (25 ± 6°, 23 ± 6°, and 24 ± 6, respectively, *P* = .063). Within each group, GI and AA displayed a negative linear correlation (R^2^ = 0.5591 for PGHOA, R^2^ = 0.4984 for MRCT, and R^2^ = 0.5829 for control group).

**Conclusion:**

CSA is a reliable parameter for predicting progression to PGHOA or MRCT, with a notable effect size. No single component of CSA predominates, and the linear correlation between GI and AA underscores a balance between these angles. Thus, CSA should indeed be considered as a “combined shoulder angle.”

The shoulder is the third most commonly affected joint by arthritis,^19^ with its incidence increasing with advancing age. This growing prevalence underscores the importance of identifying predictive risk factors for the prevention and optimal management of shoulder arthritis. The etiology of shoulder arthritis is multifactorial, primarily presenting as 2 clinical forms: concentric arthritis (primary glenohumeral osteoarthritis [PGHOA]) and eccentric arthritis, which develops secondary to massive rotator cuff tear (MRCT).

Biomechanical investigations by Gagey and Hue demonstrated that acromial morphology influences the resultant forces on the glenohumeral joint.^11^ Specifically, a short acromion was associated with increased horizontal compressive forces, while an extended acromion correlated with vertical shearing forces and overload of the supraspinatus tendon. Subsequently, Nyffeler et al^26^ introduced the “acromial index,” identifying a high index as a risk factor for cuff tears. Around the same period, Moor and Gerber proposed the “critical shoulder angle” (CSA), a novel radiographic parameter that integrates both acromial projection and glenoid inclination (GI) into a single angle.^23^ Their work showed that a CSA greater than 35° is significantly associated with rotator cuff tears, whereas a CSA less than 30° is significantly associated with concentric glenohumeral osteoarthritis.

These findings have been corroborated by multiple clinical and biomechanical studies,^15^^,^^29^^,^^31^ prompting some surgeons to perform acromioplasty with the aim of decreasing the CSA. However, the most effective method for reducing CSA to protect the rotator cuff remains unclear.^16^^,^^18^^,^^30^^,^^32^ Notably, the CSA comprises 2 distinct components: GI and the acromial angle (AA), which collectively reflect acromial projection.^10^ GI has been documented as an independent risk factor for cuff tears, a conclusion supported by several recent clinical and biomechanical investigations.^2^^,^^6^^,^^9^^,^^10^ Nevertheless, many published studies have included cases with glenoid wear, which can introduce measurement bias; worn glenoids may alter the anatomy at the superior glenoid, impacting landmark localization. Beeler et al^2^ analyzed glenoids without such wear, finding a predominant impact of glenoid coverage over GI, though their acromial measurements may have been influenced by GI itself.

Understanding the individual and combined contributions of GI and AA to the CSA could clarify which anatomical factors could be most influential in disease development. This distinction has important clinical implications, as it may guide more personalized surgical interventions or preventative strategies aimed at reducing the risk of rotator cuff tears or arthritis progression.

Our aim was to evaluate the influence of GI and AA measurements on the final CSA value in PGHOA and MRCT compared with healthy controls. We hypothesized that, within the CSA, both AA and GI have a combined influence on shoulder arthritic evolution.

## Methods

### Population

We conducted a comparative analysis among 3 groups with no evidence of glenoid wear:

− 72 patients with concentric shoulder arthritis “PGHOA group,” Walch A1-type (46 cases) and B1-type (26 cases).
− 66 patients with MRCT, “MRCT group,” Favard E0-type.
− 48 patients with healthy shoulders “control group.”

Data were obtained from a local anonymized database, with all Computed tomography (CT) scans encompassing the entire scapula. These were patients who sought medical attention for symptoms related to their osteoarthritis.

The PGHOA and MRCT groups included patients who sought medical attention for symptoms related to their osteoarthritis and who underwent CT scans for diagnosis and pre-operative planning. The PGHOA cases were either type A1 or type B1. By definition, type A1 cases exhibited minor central erosion, symmetrical subchondral condensation, and small anterior and posterior osteophytic reconstruction.^12^ As reported, a line drawn from the anterior to the posterior native glenoid rim did not intersect the humeral head in the A1 glenoid.^3^ Type B1 cases showed no bony erosion but asymmetrical posterior condensation of the subchondral glenoid bone and asymmetrical posterior glenoid cartilage wear; they also did not exhibit significant deformity that could compromise CSA measurement. The MRCTs corresponded to E0 cases, meaning there was no glenohumeral wear (chondrolysis or osteolysis). The MRCTs showed an upward displacement of the humeral head. The control group included healthy volunteers drawn from a previously published cohort^13^ (122 normal shoulders), of which 48 shoulders were selected so as to match the age range of the PGHOA and MRCT groups. The age ranges for the PGHOA, MRCT, and control groups were 65 ± 11 years, 72 ± 8 years, and 69 ± 7 years, respectively. The sex ratio was not statistically different between the groups. This study was approved by the institutional review board of the University Institute of Locomotion and Sport (Nice, France, IRB number: IRB00014528_2025_51) and conducted in accordance with the ethical standards of the 1964 Helsinki Declaration and its later amendments. Informed consent was obtained from all participants included in the study.

### Glenoid morphometrics

**Figure 1:**
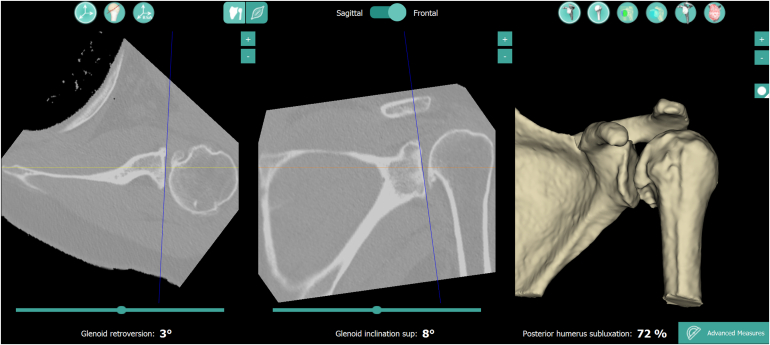
Software interface, calculation of inclination and glenoid version from CT scans data *CT*, computed tomography..

**Figure 2:**
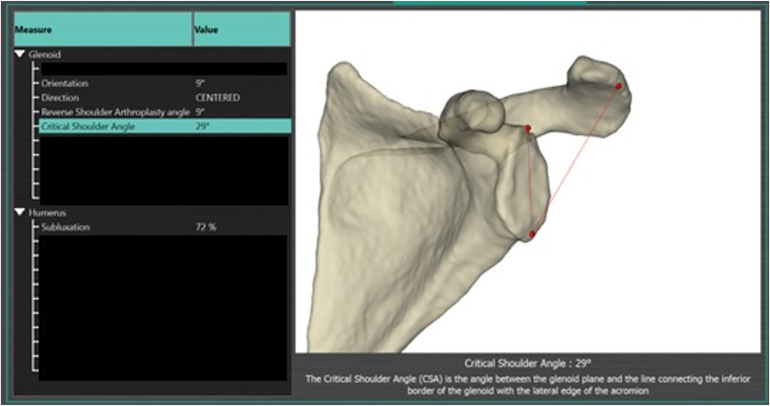
Reconstruction of CSA by the planning software. *CSA*, critical shoulder angle.

Analysis of the morphological parameters of the glenoid was performed by using an automated and validated software, Glenosys v10.4.3 (Imascap, Plouzanée, France). This software has been previously validated with high reproducibility (intraclass Correlation Coefficient >0.90) and a mean absolute measurement error below 2° for angular parameters, which is below the between-group differences reported in the present study, supporting the clinical relevance of the observed differences. Anonymized CT scan data were processed in DICOM (Digital Imaging and Communications in Medicine) format, and then automatically reconstructed into tridimensional model.^4^ The anatomical plane of the scapula was automatically computed from all the points of the entire scapula, and the transverse axis was constructed as the best-fit line to all points at the intersection of the scapular spine and the scapular body. This line has been shown to be highly robust in interindividual and intraindividual reproducibility assessments.^14^ The above data allowed defining a reference frame for the scapula to further compute automatically the GI (Fig. 1) and the CSA (Fig. 2).^5^

The CSA was calculated from:

- The most lateral point of the acromion
- The inferior glenoid point,
- The superior glenoid point

All points were projected onto the coronal plane. The CSA angle was obtained from the line connecting the superior and inferior glenoid rim points and the line connecting the inferior glenoid rim to the lateral acromial point.

The GI was measured as the angle formed by the line passing through the upper and lower points of the glenoid fossa and the line perpendicular to the transverse axis and passing through the lower point of the glenoid.

**Figure 3:**
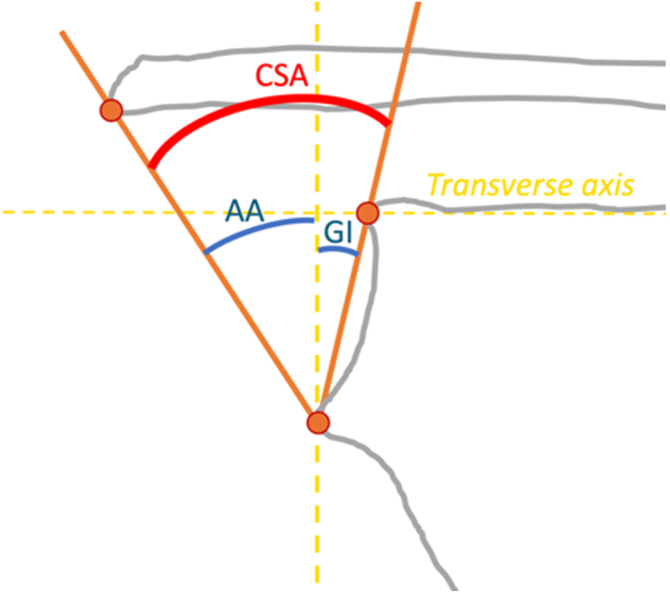
Taken in the reference frame of the transverse axis, the CSA of the scapula is the sum of 2 angles: AA and GI. Thus, AA was obtained by subtracting GI from CSA. *CSA*, critical shoulder angle; *GI*, glenoid inclination; *AA*, acromial angle.

The AA was calculated as the difference between the CSA and the GI (AA = CSA–GI)^10^ (Fig. 3).

### Statistics

Various variables were analyzed descriptively (mean, median, standard deviation, minimum, maximum). Pairwise comparisons were performed using a Tukey Kramer test. The risk α was set at 5%. A correlation test was performed to investigate the interaction of the 2 CSA components within each population. Statistical analysis was carried out with the software R^2^. Effect sizes were calculated using Cohen's d to quantify the magnitude of between-group differences independently of sample size. We interpreted d values 0.2–0.49 as a small effect, 0.5–0.79 as moderate, and ≥0.8 as large. This allows assessment of the clinical relevance of CSA, AA, and GI differences beyond statistical significance.

## Results

Table I summarizes the group comparisons. CSA differed significantly across the 3 cohorts (*P* < .05), while AA and GI showed no significant differences between the control group and either pathological group. GI was significantly greater in the MRCT group than in the PGHOA group (*P* < .05), but AA did not differ among any of the groups.

**Table I:** Comparison of CSA, GI, and AA between PGHOA, MRCT, and control groups.

Group	CSA (°)	GI (°)	AA (°)
MRCT	33 ± 4	8 ± 4.8	25 ± 5.8
Control	32 ± 3.6	7 ± 5 .1	24 ± 5.7
PGHOA	29 ± 4.2	6 ± 5.8	23 ± 6.1
MRCT/PGHOA	***P* < .05**	***P* < .05**	NS
MRCT/control	***P* < .05**	NS	NS
PGHOA/control	***P* < .05**	NS	NS

*CSA*, critical shoulder angle; *GI*, glenoid inclination; *AA*, acromial angle; *MRCT*, massive rotator cuff tear; *PGHOA*, primary glenohumeral osteoarthritis.

Bold values are statistically significant.

**Figure 4:**
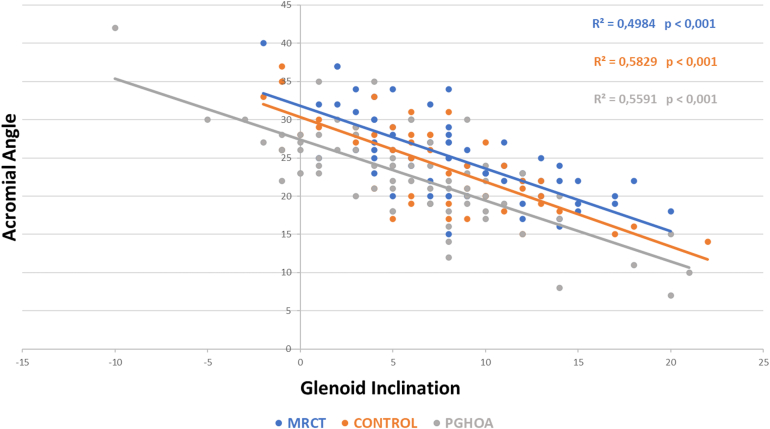
Linear correlation between acromial angle and glenoid inclination into the 3 groups. *MRCT*, massive rotator cuff tear; *PGHOA*, primary glenohumeral osteoarthritis.

**Figure 5:**
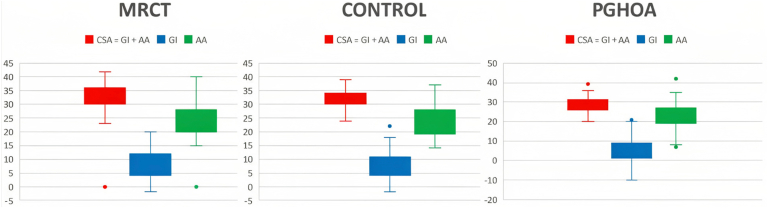
Variables distributions into the 3 groups. *CSA*, critical shoulder angle; *GI*, glenoid inclination; *AA*, acromial angle; *MRCT*, massive rotator cuff tear; *PGHOA*, primary glenohumeral osteoarthritis.

The AA and GI presented a significant and moderate linear correlation (Fig. 4). Figure 5 shows variables distribution.

Only the CSA difference between MRCT and PGHOA reached a large effect size (Cohen's d = 1.14), whereas effect sizes for both GI and AA were intermediate (d = 0.50) to small (d = 0.40), respectively. Compared to the control group, MRCT showed small effects for GI (d = 0.23) and AA (d = 0.14), while control versus PGHOA also yielded small effects for both GI and AA (d = 0.25 each).

## Discussion

The CSA represents the combined contribution of AA and GI. In our series, neither AA nor GI alone distinguished between centered (PGHOA) and eccentric (MRCT) osteoarthritis. Our main finding is that CSA emerges as a single, composite metric in which AA and GI counterbalance each other, offering a more integrative descriptor of shoulder morphology and its association with osteoarthritic patterns. Thus, CSA is in fact a “combined” shoulder angle made up of these 2 parameters, which balance each other out.

In designing this study, it was important that our populations should be made up of glenoids with no wear. Indeed, as glenoid wear could be a consequence of the existence of these morphometries, we assume that glenoid wear therefore constitutes a bias in studies working on scapular morphometric predispositions.

Moreover, selecting a normal population (control group) in the same age range as the pathological population gives us a reference for these morphometric values.

In each cohort, AA and GI were strongly and inversely correlated, reflecting the physiological balance that defines the CSA; notably, the y-intercept of each regression line closely approximated that group's mean CSA. Individually, AA and GI exhibited wide intragroup variability—indicating that neither alone predominates in pathological shoulders—whereas their sum (CSA) showed tighter standard deviation ranges and significant differences across groups. This implies that CSA abnormalities may arise from distinct AA–GI configurations (high GI/low AA or vice versa), underscoring its role as a composite measure in differentiating osteoarthritic patterns.

### Critical shoulder angle: a balance between glenoid inclination and acromial angle

Few studies have concurrently evaluated PGHOA, MRCT, and healthy cohorts. In his seminal work, Moor et al^23^ introduced the CSA against a control group but did not assess GI. Later comparisons of PGHOA and MRCT without healthy controls did report significant differences in GI and CSA.^2^^,^^9^ Beeler et al^2^ then highlighted acromial coverage as the dominant factor; however, their own analysis shows that acromial coverage is directly influenced by GI. This dependency motivated our use of the AA, as described by Engelhardt et al,^10^ which is independent of glenoid tilt. Very few studies have examined this angle. More commonly, acromial morphology has been quantified using the acromial index (AI)^26^ or the lateral AA.^1^ In a meta-analysis, Zaid et al^31^ identified 8 studies comparing cuff-tear arthropathy to healthy shoulders, most of which found significant AI differences. Yet, as Nyffeler^25^ demonstrated, AI is itself biased by GI. Including a healthy control cohort calls into question the presumed link between GI and rotator cuff tendinopathy. Indeed, several studies comparing shoulder with rotator cuff tear and healthy shoulders did not find any significant difference.^7^^,^^28^ By contrast, more recent magnetic resonance imaging-based investigations have reported significantly higher GI in patients who underwent rotator cuff repair versus those with intact cuffs.^17^^,^^22^ In the same way, few studies compare PGHOA group with healthy subjects. Gauci et al^12^ compared a PGHOA population with healthy and did not find a significant difference in GI between groups. By contrast, Smith et al^28^ found a significantly lower GI when comparing a healthy population with a PGHOA population, as did Chalmers.^8^ However, the populations of the latter 2 studies were 10 times smaller. Thus, the literature remains controversial as to the involvement of GI in shoulder arthritis. This reinforces the view that other predisposing factors such as AA could be involved and explains the discrepancies in the articles already published.

### “Action-reaction” biomechanical model behind the critical shoulder angle

**Figure 6:**
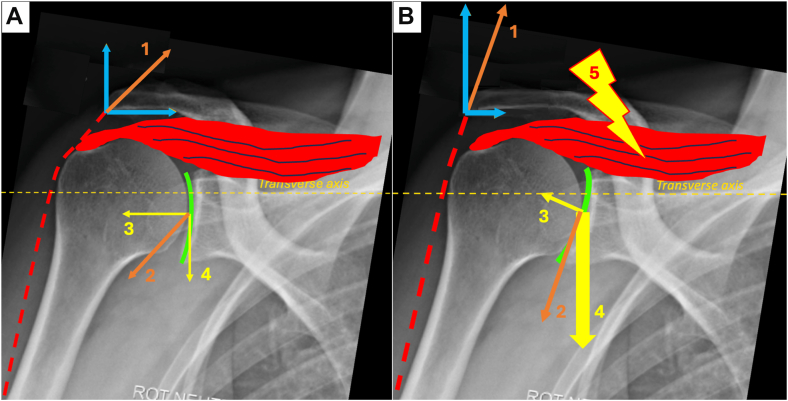
Schematic representation of the acromion/deltoid unit and the glenoid/supraspinatus unit, showing their respective local horizontal and vertical force components (*blue* and *yellow* vectors). The deltoid is represented by the *red dotted line*. The *orange* vectors represent the resultant forces of each unit; they have the same direction but opposite senses, illustrating the principle of action and reaction. This force balance enables the initiation of humeral abduction. (A): short acromion, neutral glenoid inclination = small CSA. 1: The deltoid muscle applies a resultant force that includes both horizontal (compressive) and vertical (elevating) components; 2: According to the principle of “action-reaction,” the reaction vector has the same direction and value but opposite sense, which applies to the glenoid cavity (*green*); 3: Thus, the reaction force of the glenoid cavity with neutral inclination is horizontal; 4: To counteract the upward vertical force, the supraspinatus must apply an equivalent downward vertical force. (B): large acromion, superior glenoid inclination = high CSA. 1: The deltoid muscle applies a resultant force with a significant vertical component due to the larger acromion; 2: the reaction vector applies on the glenoid with superior inclination; 3: Thus, the reaction force this time has an upward component corresponding to the upper inclination of the glenoid cavity (slope effect also contributing to the superior migration of the head); 4: As a result, the vertical forces to be counteracted are much higher; 5: The supraspinatus muscle must exert a much greater downward vertical force to compensate for the increased vertical stress in this configuration, which combines a wider acromion and a superior glenoid inclination. *CSA*, critical shoulder angle.

The biomechanical role of the CSA in both PGHOA and MRCT has been well described,^15^^,^^24^^,^^29^ with 2 key reaction-force components: shear force, which drives cranial translation of the humeral head, and compressive force, which loads the glenoid surface. Prior studies, however, have focused almost exclusively on lateral acromial overhang and have not examined the independent effect of GI, particularly at values above 5°. Extending this biomechanic framework, our work defines 2 distinct bone/muscle constructs: the acromion/deltoid unit and the glenoid/supraspinatus unit (Fig. 6). In this dual-equation model, static bony morphology determines both the magnitude and direction of muscle forces via an “action-reaction” relationship. When the AA is increased—that is, when the acromion overhangs more laterally—the deltoid's moment arm lengthens, augmenting its superior shear force on the humeral head (“action”). The compensatory “reaction” to the deltoid is an increased demand on the supraspinatus to recentralize the joint. This reaction could be further accentuated with a higher GI that redirects the joint reaction force upward and likewise elevates again the supraspinatus loading.

Following this principle, we could have found all types of conformations, including high GI with highly lateralized acromion (high AA). However, this was not the case. Indeed, we found that AA and GI were tightly but inversely correlated (r ≈ - 0.7), and neither parameter alone predicted the CSA. This lets figure out that those 2 bone/muscle units appeared to coadapt during the growth to maintain the CSA within a physiologic range. The fact that the target CSA is outside the “healthy” zone shows that this equilibrium has been shifted either upward (MRCT risk) or downward (PGHOA risk).

Clinically, this implies that an elevated CSA does not simply reflect an isolated acromial or glenoid deformity, but rather the net outcome of 2 interdependent morphologies striving to preserve glenohumeral equilibrium under muscular load. Surgically, it follows that acromioplasty and glenoid reorientation should be planned in concert to restore the composite CSA and rebalance deltoid and rotator cuff mechanics.

### Clinical perspectives

Recent systematic reviews^21^^,^^27^ have confirmed the prognostic value of the CSA for both primary rotator cuff tears and post-operative retears. Paradoxically, however, CSA has not been consistently linked to worse functional outcomes,^30^ a discrepancy that may reflect the short-term follow-up in most series. Jeong et al,^20^ for example, documented progressive functional decline in patients who sustained a recurrent tear over time after arthroscopic repair. These observations support the rationale for surgically reducing the CSA,^16^^,^^18^^,^^32^ yet standard arthroscopic acromioplasty may be insufficient in patients with a “high-GI/low-AA” profile. In such cases, persistent superior shear forces driven by a high GI undermine the benefits of lateral acromial resection. Our findings suggest that pre-operative phenotyping of a high CSA, discriminating between AA-predominant and GI-predominant contributors, could identify “good responders” to acromioplasty (those with dominant acromial overhang) and “poor responders” (those with predominant glenoid tilt). Prospective, long-term studies are needed to validate this stratification strategy.

### Strengths and limits

The very precise selection of the population (non-worn glenoid, normal shoulders with a similar age class) necessitated a reduction in the number of patients compared with our database of osteoarthritis or normal shoulders. This could explain the absence of a statistical difference of GI and AA with the control group. However, the quality of the selection enabled us to obtain clear results, and our population nevertheless includes a greater number of patients than articles reporting similar studies. Our study offers several methodological advantages over previous work. First, all measurements were obtained using automated, validated software,^4^ ensuring high reproducibility. Second, by focusing on an unworn glenoid population, we eliminated bias from osteoarthritic wear. Finally, our dataset is larger than those in the literature—particularly for the rare B1 glenoid type and for elderly normal shoulders—providing a robust control group.

## Conclusion

CSA is a reliable parameter for predicting progression to PGHOA or MRCT, with a notable effect size. No single component of CSA predominates, and the linear correlation between GI and AA underscores a balance between these angles. Thus, CSA should indeed be considered as a “combined shoulder angle.”

## Disclaimers:

Funding: No funding was disclosed by the authors.

Conflicts of interest: Marc-Olivier Gauci is a consultant for Imascap and Medacta. Any additional authors, their immediate families, and any research foundations with which they are affiliated have not received any financial payments or other benefits from any commercial entity related to the subject of this article.

## References

[1] Banas M.P., Miller R.J., Totterman S. (1995). Relationship between the lateral acromion angle and rotator cuff disease. J Shoulder Elbow Surg.

[2] Beeler S., Hasler A., Götschi T., Meyer D.C., Gerber C. (2019). Critical shoulder angle: acromial coverage is more relevant than glenoid inclination. J Orthop Res.

[3] Bercik M.J., Kruse K., Yalizis M., Gauci M.-O., Chaoui J., Walch G. (2016). A modification to the Walch classification of the glenoid in primary glenohumeral osteoarthritis using three-dimensional imaging. J Shoulder Elbow Surg.

[4] Boileau P., Cheval D., Gauci M.-O., Holzer N., Chaoui J., Walch G. (2018). Automated three-dimensional measurement of glenoid version and inclination in arthritic shoulders. J Bone Joint Surg Am.

[5] Bouaicha S., Ehrmann C., Slankamenac K., Regan W.D., Moor B.K. (2014). Comparison of the critical shoulder angle in radiographs and computed tomography. Skeletal Radiol.

[6] Bouaicha S., Kuster R.P., Schmid B., Baumgartner D., Zumstein M., Moor B.K. (2020). Biomechanical analysis of the humeral head coverage, glenoid inclination and acromio-glenoidal height as isolated components of the critical shoulder angle in a dynamic cadaveric shoulder model. Clin Biomech.

[7] Chalmers P.N., Beck L., Miller M., Kawakami J., Dukas A.G., Burks R.T. (2020). Acromial morphology is not associated with rotator cuff tearing or repair healing. J Shoulder Elbow Surg.

[8] Chalmers P.N., Miller M., Wheelwright J.C., Kawakami J., Henninger H.B., Tashjian R.Z. (2021). Acromial and glenoid morphology in glenohumeral osteoarthritis: a three-dimensional analysis. JSES Int.

[9] Daggett M., Werner B., Collin P., Gauci M.-O., Chaoui J., Walch G. (2015). Correlation between glenoid inclination and critical shoulder angle: a radiographic and computed tomography study. J Shoulder Elbow Surg.

[10] Engelhardt C., Farron A., Becce F., Place N., Pioletti D.P., Terrier A. (2017). Effects of glenoid inclination and acromion index on humeral head translation and glenoid articular cartilage strain. J Shoulder Elbow Surg.

[11] Gagey O., Hue E. (2000). Mechanics of the deltoid muscle. A new approach. Clin Orthop.

[12] Gauci M.-O., Athwal G.S., Sanchez-Sotelo J., Chaoui J., Urvoy M., Boileau P. (2021). Identification of threshold pathoanatomic metrics in primary glenohumeral osteoarthritis. J Shoulder Elbow Surg.

[13] Gauci M.-O., Deransart P., Chaoui J., Urvoy M., Athwal G.S., Sanchez-Sotelo J. (2020). Three-dimensional geometry of the normal shoulder: a software analysis. J Shoulder Elbow Surg.

[14] Gauci M.-O., Jacquot A., Boux de Casson F., Deransart P., Letissier H., Berhouet J. (2022). Glenoid inclination: choosing the transverse axis is Critical-A 3D automated versus manually measured study. J Clin Med.

[15] Gerber C., Snedeker J.G., Baumgartner D., Viehöfer A.F. (2014). Supraspinatus tendon load during abduction is dependent on the size of the critical shoulder angle: a biomechanical analysis. J Orthop Res.

[16] Girard M., Colombi R., Azoulay V., Laumonerie P., Martel M., Mansat P. (2020). Does anterior acromioplasty reduce critical shoulder angle?. Orthop Traumatol Surg Res OTSR.

[17] Gulcu A., Aslan A., Dincer R., Özmanevra R., Huri G. (2022). Relationship between diagnostic anatomic shoulder parameters and degenerative Rotator Cuff tears: an MRI study. Orthop J Sports Med.

[18] Hardy V., Rony L., Bächler J., Favard L., Hubert L. (2021). Does anterior arthroscopic acromioplasty modify critical shoulder angle?. Orthop Traumatol Surg Res OTSR.

[19] Ibounig T., Simons T., Launonen A., Paavola M. (2020). Glenohumeral osteoarthritis: an overview of etiology and diagnostics. Scand J Surg.

[20] Jeong H.J., Nam K.P., Yeo J.H., Rhee S.-M., Oh J.H. (2022). Retear after Arthroscopic Rotator Cuff repair results in functional outcome deterioration over time. Arthrosc J Arthrosc Relat Surg.

[21] Liu T., Zhang M., Yang Z., Zhang B., Jiang J., Yun X. (2023). Does the critical shoulder angle influence retear and functional outcome after arthroscopic rotator cuff repair? A systematic review and meta-analysis. Arch Orthop Trauma Surg.

[22] Maalouly J., Tawk A., Aouad D., Nour H.A., Saidy E., Abboud G. (2020). Is there an association between glenoid parameters and rotator cuff tears and the influence of gender: a retrospective study on a Middle Eastern population. Int J Surg Case Rep.

[23] Moor B.K., Bouaicha S., Rothenfluh D.A., Sukthankar A., Gerber C. (2013). Is there an association between the individual anatomy of the scapula and the development of rotator cuff tears or osteoarthritis of the glenohumeral joint?: a radiological study of the critical shoulder angle. Bone Joint J.

[24] Moor B.K., Kuster R., Osterhoff G., Baumgartner D., Werner C.M.L., Zumstein M.A. (2016). Inclination-dependent changes of the critical shoulder angle significantly influence superior glenohumeral joint stability. Clin Biomech Bristol Avon.

[25] Nyffeler R.W., Meyer D.C. (2017). Acromion and glenoid shape: why are they important predictive factors for the future of our shoulders?. EFORT Open Rev.

[26] Nyffeler R.W., Werner C.M.L., Sukthankar A., Schmid M.R., Gerber C. (2006). Association of a large lateral extension of the acromion with rotator cuff tears. J Bone Joint Surg Am.

[27] Oladimeji A.E., Amoo-Achampong K., Ode G.E. (2024). Impact of critical shoulder angle in shoulder pathology: a current concepts review. JSES Int.

[28] Smith G.C.S. (2022). A prospective observational case control study investigating the coronal plane scapular morphological differences in full-thickness posterosuperior cuff tears and primary glenohumeral osteoarthritis. J Shoulder Elbow Surg.

[29] Viehöfer A.F., Snedeker J.G., Baumgartner D., Gerber C. (2016). Glenohumeral joint reaction forces increase with critical shoulder angles representative of osteoarthritis-A biomechanical analysis. J Orthop Res.

[30] Yang S., Pang L., Zhang C., Wang J., Yao L., Li Y. (2024). Lower reoperation rate and superior patient-reported outcome following Arthroscopic Rotator Cuff repair with concomitant acromioplasty: an updated systematic review of randomized controlled trials. Arthrosc J Arthrosc Relat Surg.

[31] Zaid M.B., Young N.M., Pedoia V., Feeley B.T., Ma C.B., Lansdown D.A. (2019). Anatomic shoulder parameters and their relationship to the presence of degenerative rotator cuff tears and glenohumeral osteoarthritis: a systematic review and meta-analysis. J Shoulder Elbow Surg.

[32] Zhang M., Yang Z., Zhang B., Liu T., Jiang J., Yun X. (2022). Does the critical shoulder angle decrease after acromioplasty? A systematic review and meta-analysis. J Orthop Surg.

